# Children’s Active School Travel: Examining the Combined Perceived and Objective Built-Environment Factors from Space Syntax

**DOI:** 10.3390/ijerph18010286

**Published:** 2021-01-02

**Authors:** Ayse Ozbil, Demet Yesiltepe, Gorsev Argin, Greg Rybarczyk

**Affiliations:** 1 Department of Architecture and Built Environment, Faculty of Engineering and Environment, Northumbria University, Newcastle upon Tyne NE1 8ST, UK; demet.yesiltepe@northumbria.ac.uk; 2 Department of Urban and Regional Planning, İstanbul Technical University, İstanbul 34367, Turkey; arging@itu.edu.tr; 3 College of Arts and Sciences, University of Michigan-Flint, Flint, MI 48502, USA; grybar@umich.edu; 4 The Michigan Institute for Data Science (MIDAS), Ann Arbor, MI 48108, USA; 5 The Centre for Urban Design and Mental Health, London SW9 7QF, UK

**Keywords:** active school travel, street connectivity, public health, childhood obesity, GIS, space syntax, nominal regression, İstanbul, Turkey

## Abstract

Increasing active school travel (AST) among children may provide the required level of daily physical activity and reduce the prevalence of obesity. Despite efforts to promote this mode, recent evidence shows that AST rates continue to decrease in suburban and urban areas alike. The aim of this research study, therefore, is to facilitate our understanding of how objective and perceived factors near the home influence children’s AST in an understudied city, İstanbul, Turkey. Using data from a cross-sectional sample of students aged 12–14 from 20 elementary schools (*n* = 1802) and consenting parents (*n* = 843), we applied a nominal logistic regression model to highlight important predictors of AST. The findings showed that street network connectivity (as measured by two novel space syntax measures, metric reach and directional reach) was the main deciding factor for active commuting to school, while parents’ perceptions of condition of sidewalks and shade-casting street trees were moderately significant factors associated with AST. Overall, this study demonstrated the significance of spatial structure of street network around the homes in the potential for encouraging AST, and more importantly, the need to consider objective and perceived environmental attributes when strategizing means to increase this mode choice and reduce ill-health among children.

## 1. Introduction

Physical inactivity among children is becoming increasingly prevalent in many developing countries, including Turkey, which has faced rapid economic and social development, urbanization, and industrialization [1]. The World Health Organization [2] recommends that children should undertake at least 60 minutes of moderate-to-vigorous physical activity each day. In Turkey, 56.2% of 12–14-year-olds do not meet the recommendation [3]. This has key implications for children’s health conditions, including mental wellbeing [4], obesity [5], diabetes, cardiovascular diseases, and asthma [6,7]. Active school travel (AST) has important potential for providing the required level of daily physical activity of children [8]. Promoting AST has also shown to positively impact children’s physical health (i.e., reduced BMI and increased mental well-being among children actively commuting to school [9,10,11]). However, in recent years many medium- and high-income countries throughout Europe and globally have seen a decline in the number of children walking to school [12,13].

AST can be defined as any type of human-powered transportation, such as walking, and bicycling [14]. Children’s AST is complex and remains poorly understood. Findings have shown that personal factors, and perhaps more important, parental influence weigh heavily on this mode choice. Childhood traits such as: genetic predisposition [15], socio-economic status, and dietary habits negatively affect AST [16]. Children’s demographics also weigh heavily on AST. Past works suggest that gender [17,18], age [19,20], and ethnicity [21,22] all contribute to AST. Regarding parents, both their personal attributes and attitudes (e.g., parents’ own history of transport to school, perceptions of the importance of physical activity in their lives) affect children’s AST [18,23,24]. Parental concerns about the environmental conditions along the walk to school include general difficulty of walking, perceived travel distance, and traffic/crime danger [25,26,27]. Other parental concerns include presence of physical barriers (e.g., highway, busy road), quality of the walking environment (e.g., shaded by trees, quiet walking environments, well maintained and clean environments), land use (e.g., office buildings, large parking lots, vacant lots) [28], weather condition [29], and parents’ perceptions of their neighborhood (e.g., friendly people in the neighborhood, good local shops) [30]. Parents’ perceptions of their child’s road-crossing ability [31] were also shown to influence children’s mode of commuting to school. The majority of these factors have been linked to the likelihood of children choosing AST, even when controlling for a multitude of walkability assessments [32]; however, the findings remain mixed. For example, DeWeese et al. [33] and Trang et al. [34] did not find perceptions of sidewalk conditions as predictors of AST. Parents’ attitudes regarding street connectivity have also been shown to influence the likelihood of children’s active commuting to school [32]. The socio-economic status (SES) and educational attainment of the parents influence children’s AST as well. For example, a plethora of past studies has indicated that employment status, car ownership, parental education, and income [17,20,35,36] have been found to influence rates of AST. The evidence remains indeterminate on this too. Most studies found an inverse relationship between car availability and rates of walking/active travel to school among children [26,37,38,39]. Other research contends that families with reduced SES live in dense urban areas and have shorter distances for school trips [40], and hence, would live within a shorter distance to school. On the contrary, it has also been posited that less affluent households may have been dislocated to the periphery of the city [41] with less connected streets and increased distances to schools. Overall, evidence points to the dynamic relationship between parental attitudes, geography (i.e., where one lives), SES, and children’s AST.

A significant number of past studies have shown that geography is an important factor; meaning that where one lives (i.e., urban versus suburban zones) influences this activity [42,43]. The strongest geographical predictor of AST is distance [44,45]. Previous correlational studies have indicated that increased distance is associated with increased walking times and therefore decreased active travel modes to school [46,47]. Contrastingly, others have demonstrated a significant inverse relationship between the distance between home and school and the probability of AST within 1600 m (1 mile) of the school [48]. Not only is the distance between home and school important for predicting children’s AST, but neighborhood composition is also key [44].

A growing body of research has demonstrated that neighborhoods characterized by land-use and street network design can have positive and negative impacts on children’s activity levels [49,50]. For instance, children living in pedestrian-friendly environments have higher proportions of daily walking [13]. Land-use mix has shown to be a key determinant of active travel rates among children [37], with a greater diversity of land-uses within walking range resulting in a greater number of available destinations to walk to. Researchers have suggested that commingling of a variety of activities, such as residences, shops, restaurants, and offices, supports walking mode choice [50,51,52,53]. However, the relationship remains mixed. Some studies found a positive relationship between land-use mix and rates of active travel to school [32,35,37,54], whereas others found the opposite effect [20,38]. The majority of studies found land-use mix to have an insignificant impact on rates of active travel to school [55,56,57].

In the last decade, research on AST has emphasized the importance of street network design [58]. Several previous works have revealed that shorter block sizes, or densely connected street network layouts (i.e., increased density of intersections), may increase walking rates and allow for more frequent and longer walks [59,60,61,62,63]. Despite these findings, the magnitude and direction of influence remains ambiguous. One reason is the narrow definition of the built environment. Previous research has not adequately addressed street design when predicting children’s AST. In other words, standard measures of street layout lack in systematically evaluating the spatial structure of urban street networks at various scales (local neighborhood scale vs. larger urban scale structure). Spatial structure may be defined as the collection of streets and street segments through certain visual alignments and hierarchies. Space syntax has been proven a valid means to empirically assess these relationships and has demonstrated important relationships to pedestrian movement [64]. This method suggests that the structure of an urban street network, as defined by the connectivity hierarchy measured by direction changes, plays an important role in pedestrian travel [65,66]. Earlier research demonstrated that streets which are accessible from their surroundings with fewer direction changes tend to attract higher densities of pedestrian flows [67,68]. Additionally, other studies have shown that increased street network connectivity, as measured through syntactic measures, is significantly related to higher recreational walking rates [69], walking rates to transit stops [70], AST [57], and decreased prevalence of childhood obesity [71].

Considering the research above, the current investigation contributes to the growing body of work on understanding means to increase physical activity levels among children by examining children’s AST. While there is a large amount of research into the school journey, a key limitation is that few studies have assessed both objective and perceived measures of walkability as explanatory factors for walking, in general [62,63,72] and for AST, in particular [73,74]. This necessitates further enquiry. The main goal of this study, and hence research contribution, is to assess the effect of built environment conditions, coupled with neighborhood perceptions, on children’s AST. Another key research limitation in prior AST studies has been the treatment of the built environment. Space syntax has proven to be a valid means of quantifying how urban design relates to movement potentials [66,75]; however, it has received little attention in the related literature. We thus implement novel measures of street connectivity obtained from space syntax, which overcomes the limitations of several past connectivity measures used in AST studies. Furthermore, there is minimal research on AST among children in the country of Turkey, where promoting this mode has become a national priority [76]. Understanding children’s AST in this region of the world is needed as the rates of childhood obesity and mental health problems among children and adolescents are presently mirroring many other industrialized nations.

## 2. Materials and Methods

### 2.1. Study Area Context

The study area in this research is İstanbul, Turkey, and select neighborhoods (i.e., districts) within. The city is the largest city in Turkey and contains an area of 5461 km^2^ and an estimated population of 15,067,724 [77]. The street network of the city differs in various neighborhoods, from traditional grid-iron geometry to loop and cul-de-sac street patterns. The city is also interesting in that neighborhoods of both urban center and general urban tend to have a mixture of these different types of street network layouts. The residential street pattern design in the city was shaped by the mode of transportation of the day, the current models of urban planning, and the recent growth of the city towards its periphery. Public transportation system is quite extensive and cheap (as compared to other European countries) while bicycling among the youth and general population is rather limited. In terms of travel modes to school, the 2012 İstanbul household survey shows that 68.8% of home-based school trips were made on foot; walking is also highly preferred by İstanbulites for home-based other trips (59.8%), non-home-based trips (31.3%), and home-based work trips (23.7%) [78]. The survey also indicates that private automobile use for non-home-based trips is the most popular mode, as it comprised 40.4% of the sample, followed by the home-based work trips (20.6%), home-based other trips (14.9%), and home-based school trips (3.2%).

Neighborhoods were selected based on their variability in objectively measured walkability and household education, based on three criteria shown to be associated with walking behavior in the literature:

- Representation of areas in similar numbers from neighborhoods with high, medium, and low education rates (high school degree and above) according to the 2012 data of TUIK (Turkish Statistical Institute),
- Inclusion of districts with a population density above 10,000 (person/km) according to İstanbul Metropolitan Municipality 2012 data, and
- Full diversity of average street connectivity levels (high, medium, low)—measured by metric reach, which calculates the total street length accessible within the threshold starting from the mid-point of each street segment—in 1600 m radius of schools.

**Figure 1:**
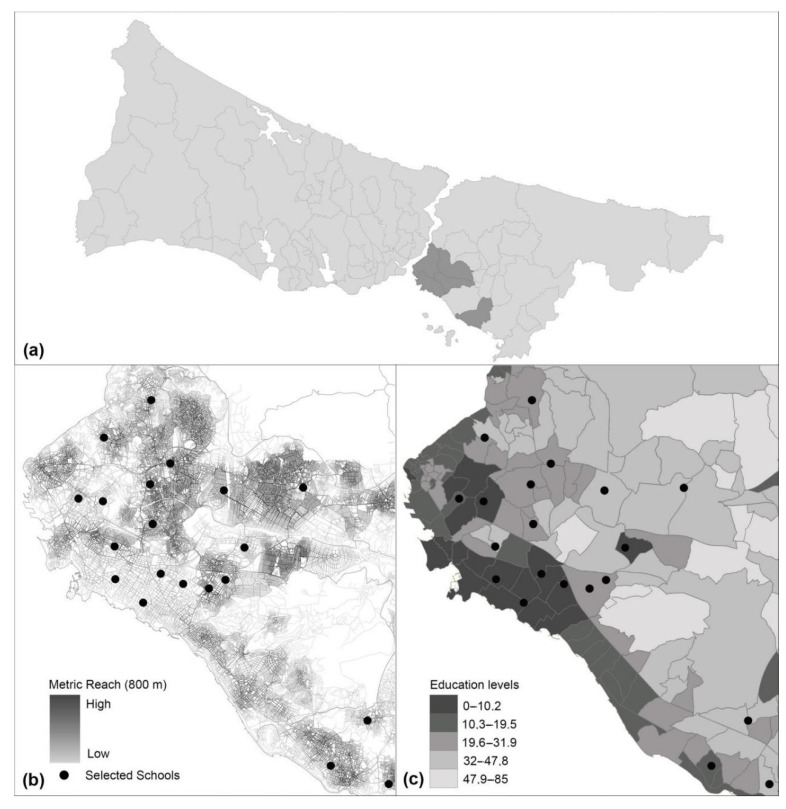
Location of surveyed schools. Maps are color-coded based on (**a**) selected districts, (**b**) metric Reach (800 m) values of the street network, and (**c**) the district-based average education levels (high school).

The 20 elementary schools selected according to these criteria are distributed over 5 districts (Kadıköy, Üsküdar, Kartal, Ataşehir, and Ümraniye) (Figure 1) located in the Anatolian part of the city. Kadıköy and Üsküdar are central-city districts, and due their historic background, they have both organically planned and grid-like street network structures. Ataşehir, which became a district in 2008, is a contemporary in-town suburb with high-end residential gated-communities and office skyscrapers while Ümraniye and Kartal are general urban districts. The underlying reason for studying the Anatolian part is due to the different urban patterns dominating each continent. The European part is mostly dominated by high-rise mass housing, service, and commercial land-uses, whereas the Anatolian part reflects mostly a residential character with mixed land-uses prevailing in the central parts. Although the selected areas represent a small cross-section of the entire city, the sum of their population equals one-sixth of İstanbul’s total population.

### 2.2. Sampling

The sampling design in this research was cross-sectional. Students in grades 6, 7, and 8 (aged 12–14) at these schools (~100 students per school, *n* = 1802) were selected randomly based on the availability of their class schedules to take part in the study. In consultation with the school principals, teachers of 12–14 age groups volunteered to have their classrooms participate in the study. The study protocol was approved by Ethics Commission, Özyeğin University (Ethics ID 2013/03) and relevant permissions were granted by the İstanbul Directorate of National Education (ID 59090411/605/2329961). Written informed parental consent and the assent of the children were obtained. The study was introduced to students in their classes where their class teachers were present. A letter explaining the study accompanied with the parental survey, providing background and contact information, was sent to parents via students. Parents were given 3 weeks to return the questionnaire/consent form to the school. Upon receipt of the parental questionnaires and parental consent forms, the names of participants were coded to help maintain confidentiality. Student questionnaires were conducted after the receipt of parental consent and children’s assent during a second visit to the schools. Student surveys were conducted face to-face, whereas parental questionnaires were sent to/collected from parents via children.

The surveys used in this study are cross-sectional and obtained from parents’ and children’s questionnaires conducted between May 2014 and May 2015. The participating children were asked to complete a questionnaire that asked for their full address, age, primary mode of commuting to and from school, and if not actively commuting, main reasons for not walking to school. Since we were more interested in the built environment barriers, rather than enablers, in influencing children’s decision to walk to/from school and in line with past research [79,80], we did not ask children their reasons for walking (i.e., environmental enablers). As argued by Lu et al. [81], increasing walk-to-school rates is possible if researchers can identify the barriers preventing children from walking to school. Parents of participating children were also invited to answer questions regarding their income, car ownership, education, and the primary reasons for not allowing their children to walk to school as well as questions with regard to their home-neighborhood. By asking both students and parents about underlying reasons for not walking and not allowing to walk to/from school, respectively, the perception of both groups with regard to AST was captured. Parents with more than one child in the sample were eliminated. The return rate of parental questionnaires was 62% and uncompleted questionnaires (*n* = 275) were not included in further analyses, which left a total of 843 cases available to analyze. Therefore, the final student dataset included the answers of 1802 pupils, of whom 33% were from the 6th grade (approximately 12 years old) and 31% from 8th grade (approximately 14 years old), while the final merged dataset (students and parents) included 843 cases.

### 2.3. Measures

The measures used in this research are depicted in Table 1. The aforementioned surveys provided many important indicators regarding children’s demographic and parental socioeconomic characteristics and were incorporated into this study. Students were asked to report their primary mode of access for school-bound and home-bound trips (e.g., walking, car, school bus, public transport), and the responses were categorized into two dichotomous variables: “walk either way” and “walk both ways”. Since cycling did not exist as mode choice among this sample, only walking was regarded as mode of AST. Unfortunately, in İstanbul, cycling as a transport mode is not prevalent due to the limited bicycle infrastructure, heavy traffic, and hilly terrain. According to the İstanbul Household Survey conducted in 2012, the share of bicycle usage in daily trips in İstanbul was only 0.1% [78]. Similarly, since the rate of using public transport to/from school was negligible (2.8% and 3.8% for school-bound and home-bound trips, respectively) in this sample and since we did not collect data on the full journey of these students (i.e., whether they walked as part of their commute), we chose not to include these students as part of the “active commuting” sample. 

**Figure 2:**
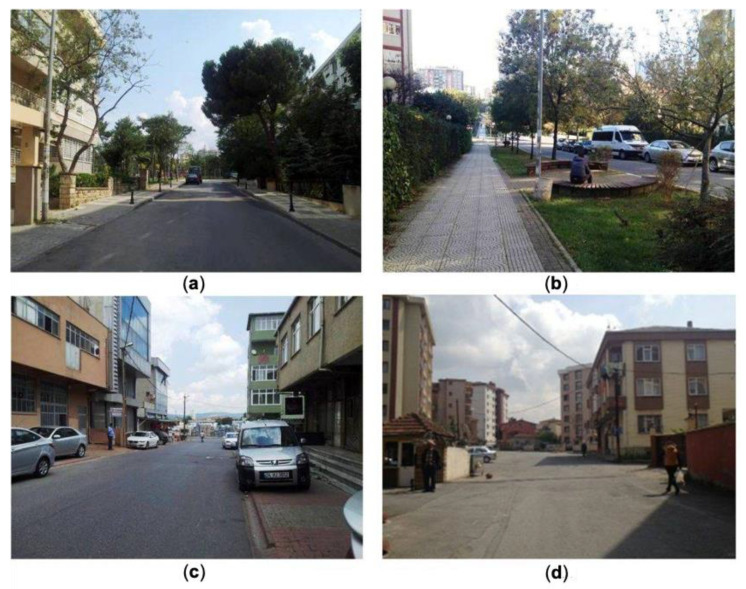
Typical streets within home-neighborhoods in (**a**) Kadıköy, (**b**) Ataşehir, (**c**) Ümraniye, and (**d**) Kartal.

Information regarding children’s demographic and parental socioeconomic characteristics was also used in this research. In addition, parents reported their level of agreement with a set of statements, using a 5-point scale (“strongly disagree”, “disagree”, “neutral”, “agree”, and “strongly agree”), regarding their perception of home-neighborhood. These statements were adapted from the validated Neighborhood Environment Walkability Scale (NEWS) [82], modified to fit into the Turkey context, translated into Turkish, and validated through a pilot study. This scale assessed environmental characteristics considered to be associated with physical activity, including (a) accessibility and streets, (b) access to urban services, (c) architecture, (d) green spaces, (e) safety, and (f) maintenance. Since the focus of this paper is on identifying the effects of street-related attributes on AST, we included “accessibility and streets” (e.g., sidewalks) and “street network layout” in the study. Figure 2 depicts a typical streetscape within home-neighborhoods in four of the study districts.

Students who did not actively commute to school (*n* = 580) were asked to report their primary reasons for inactive commuting through a seven-option multiple-choice question, including an open-ended option to include any reasons that fell outside the multiple-choice answers. Parents were also asked to report their main underlying reasons for not allowing their children to walk to school, similarly through a five-option multiple-choice question with an open-ended option. Both groups were allowed to select multiple answers. These questions were adapted from studies using a similar approach [83,84].

### 2.4. GIS-Based and Space Syntax Measures

The GIS analysis in this research relied on ArcGIS software (version 10.2.2. Environmental Systems Research Institute (ESRI): Redlands, CA, USA). The first step was to geocode the street addresses of all study participants. Since 0.5 mile (800 m) is considered a reasonable distance for active commuting [85], home-neighborhood buffers based on this distance were created using GIS. Parcel-level land-use densities (residential, retail, and recreational) were summarized within each home-neighborhood area.

**Figure 3:**
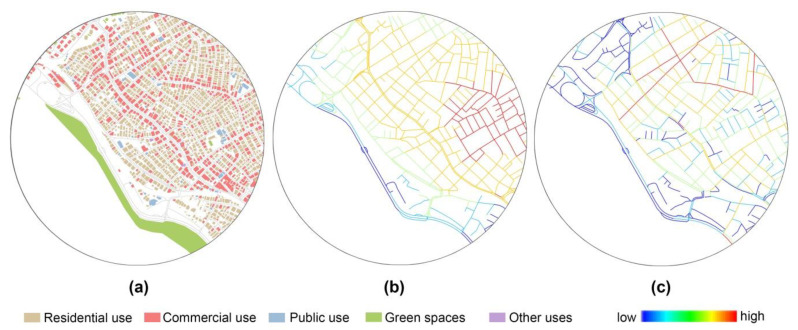
Evaluation of a home-neighborhood (800 m circular buffer) through (**a**) land-use patterns, (**b**) metric reach (800 m), and (**c**) 2-directional reach (10°).

Street network configuration of the Anatolian part was analyzed using space syntax. In particular, two key measures of connectivity were produced: metric reach and directional reach [86]. In metric reach analysis, the density of street network is captured by measuring the total street length accessible from each street segment up to a parametrically specified metric distance threshold. Directional reach measures the total street length accessible from each street segment up to a certain number of direction changes, parametrically defined via a threshold angle. Metric reach was computed for an 800-m walking distance threshold. Directional reach was computed for two direction changes subject to a 10° angle threshold. The 10° angle threshold was selected to ensure that the analysis was sensitive to the distinction between linear and curvilinear systems. Computing directional reach for two direction changes measures the extent to which a street segment is embedded within the surrounding street network in terms of directional distance. These refined measures of street connectivity can differentiate between well- and less well-connected road segments and streets within a given area, whether it is a grid, a curvilinear pattern or a cul-de-sac. Figure 3 displays 800-m circular buffers of one home-neighborhood evaluated by metric and directional reach as well as parcel-based land-uses.

### 2.5. Analysis

The analysis in this study is threefold. Firstly, we present descriptive analysis for all students (*n* = 1802) who participated in the study. Secondly, parent/child perceptions discouraging AST were analyzed through the responses of students who did not walk to school and their parents who did not allow them to actively commute (*n* = 580) among the whole sample. Lastly, associations with walking to/from school were examined through a nominal logistic regression model in which the macro-scale attributes of the home-neighborhood and perceived street-level environmental features were analyzed simultaneously. In order to control for the effects of distance to school, only students residing within 1600 m of their schools (*n* = 749) were included in the model since the literature has shown 1 mile (1.6 km) as the walking threshold between origins and destinations [17].

## 3. Results

### 3.1. Descriptive Analysis

Table 2 shows the composition of the survey participants by school and district; number of children and mean age are reported per gender group while walk mode shares (home-bound and school-bound) are reported based on school. Of the 1802 children, 907 were male and 895 were female. The average age was 13.4. The average walk mode share for school-bound and home-bound trips (both ways) was 65.8% while it was 76% for either way (home-bound and/or school-bound) for the entire sample. The average walk mode share, both ways and either way, per school was highest for School S03 (79.5% and 97.7%, respectively) in Ümraniye while it was the lowest for School S04 (34.6% and 39.7%, respectively) in Kadıköy and for School S03 (37.2% and 44.2%, respectively) in Ataşehir.

The commuting mode shares to and from school among the sample are reported in Table 3. It is evident that the majority travelled to/from school by walking, followed by car and school shuttle. The table also indicates that public transport and cycling mode shares were negligible.

### 3.2. Parent/Child Perceptions Discouraging AST

**Figure 4:**
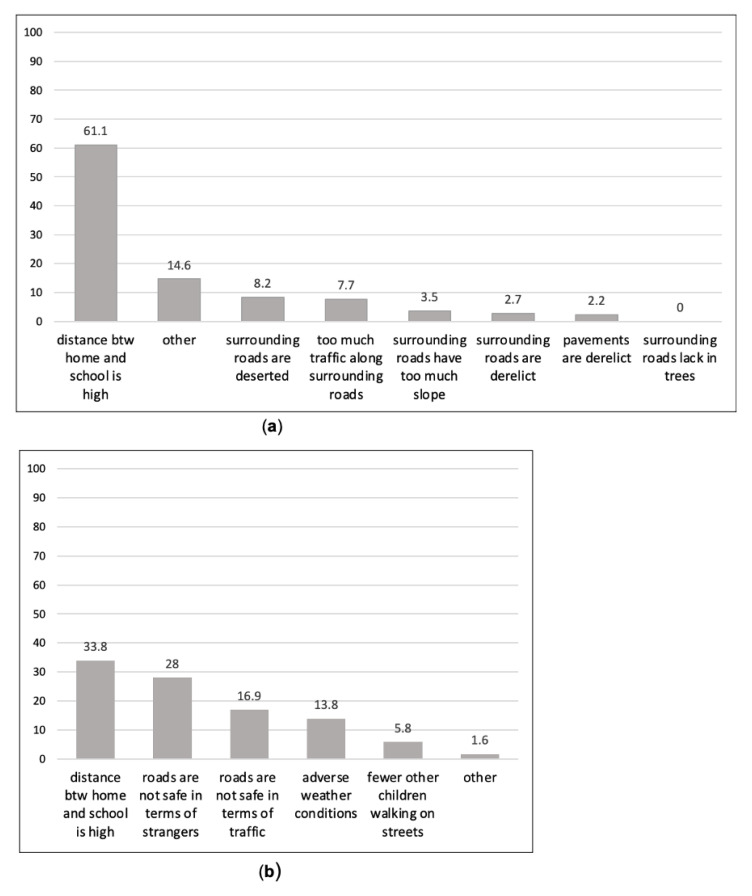
Responses of participants: (**a**) students’ primary reasons for not wanting to AST, and (**b**) parents’ reasons for not allowing AST.

According to students who did not choose AST (*n* = 580), the main perceived barrier was distance between home and school (61.1%) (Figure 4a). The mean, median, and range of actual network distance between participant’s home and school for those who perceived the home-school distance “too far” were 3.4 km, 2.6 km, and 16.8 km respectively. We also discovered other important barriers to AST. Noteworthy factors included deserted roads (8.2%), heavy traffic flows (7.7%), neglected roads (2.7%), excessive slope (3.5%), and disorderly pavements (2.2%). The lack of trees along the sidewalks was not found to be a barrier to AST. We can infer that environmental safety (physical and social), rather than the road/pavement maintenance and aesthetics, was more effective in encouraging AST.

When asked about the reason for not allowing their children to walk to school, parents listed distance (33.8%), roads being unsafe in terms of crime (e.g., strangers) (28%), traffic (16.9%), bad weather (13.8%), and relatively small number of other children walking along the streets (5.8%) as the main barriers (Figure 4b). The mean, median, and range of network distance between participant’s home and school for parents considering the home-school distance “too far” were 2.7 km, 2.1 km, and 10.5 km, respectively.

When students’ open-ended responses are analyzed, increased distance to school is central to many students’ perceived barriers to AST:

“my home is too far away to my school”; “the route is too long, and I become tired”.

Another common theme is the fear of stranger-danger:

“when returning from school, it is dark, and I am afraid”; “there is no one on the streets”; “the route between home and school is dangerous—too deserted”; “there are many tramps/bad people along the way”; “in the morning the roads are very deserted”; “I can be abducted”.

The condition of sidewalks along the route was also shared by a number of students:

“sidewalks are too narrow”; “sidewalks are poorly maintained, with lots of pots”; “there are cars parked on the sidewalks”; “the construction works spill onto the sidewalks, blocking the way”

Furthermore, some students reported that they were willing to walk to school, but that their parents would not allow them.

“my parents do not allow”; “my dad thinks walking is dangerous”; “I really want to walk but my mom does not allow it”

The findings here echo earlier studies highlighting that parents are the “gatekeepers” in terms of AST among children [87,88]. Parental barriers transcribed from the open-ended responses are in agreement with those of the students. The primary perceived barrier to AST is distance and secondary to this are traffic safety and crime.

### 3.3. Associations with Walking to/from School

Nominal logistic regression modeling was used to explore the association of the active commuting outcome (walked to/from school versus did not walk to/from school, either way), as the nominal dependent variable, and the macro-scale attributes of the home-neighborhood (land-use and street network connectivity) and parents’ perceived street-level environmental features, as the independent variables. Nominal logistic modeling was used since the outcome variable was a categorical (or nominal, i.e., has two categories with no intrinsic ordering) response variable. The model was adjusted for child gender, parental education, and household car ownership—which served as a proxy variable for socioeconomic status since the majority of parents did not report their income. The statistic outcomes indicated a high correlation between car ownership rates and income levels in İstanbul [89], with households having no cars and owning one car representing 63.6% and 33.9%, respectively [78].

As suggested by Basolo and Strong [90], to better dichotomize the variables regarding the street-level environmental perceptions of parents, response categories of “highly agree” and “agree” were merged into a single category, “agree”, and all other responses (i.e., disagreement and neutral selections) were categorized as “other”. The relationship between the variables was also examined to check for collinearity. The district identifier was included as a fixed effect in the final model to capture the effects of different districts on the choice to walk to/from school. The regression model was conducted in JMP (JMP^®^, Version 12.2.0 SAS Institute Inc., Cary, NC, USA, 1989–2019).

The estimated coefficients of the variables, standard errors, and Wald chi-square (χ^2^) statistics, with the corresponding significance levels, are reported in Table 4. To determine the most influential attributes, Wald χ2 statistics were used, similar to other studies [91]. Variables with higher Wald χ2 values are considered to have a greater contribution to the model, hence are considered to be the most important correlates [92]. The regression model shows a statistically significant result (*p* = 0.00), and the goodness-of-fit test shows that the model adequately fits the data (*p* > 0.9). Variables that had a 95% confidence interval or higher (i.e., *p* < 0.05) were considered as significantly correlated with walking to/from school.

Increased parental education (i.e., both parents having college degree or above) and household car ownership (having one car vs. no car) were significantly associated with AST. Although this positive relationship found between increased car ownership and walk mode choice is contrary to some earlier findings [44,93], it might point to the fact that parents with increased car ownership, hence, income, may afford to live in neighborhoods with increased walkability (e.g., higher streets density and pedestrian-oriented streetscapes). Based on the Wald χ^2^ values, density of streets around the home, as measured by metric reach (Wald χ^2^ = 20.32), was the strongest correlate of walking to school. Surprisingly, residential density (Wald χ^2^ = 20.17) and retail density (Wald χ^2^ = 15.34) were negatively correlated with AST. This may be due to the increasing levels of automobile traffic associated with increased land-use densities, which can pose dangerous conditions for walking. [94]. Notably, the space syntax metric, 2-directional reach (Wald χ^2^ = 4.79), showed modest associations with AST. We can infer from this that greater directional accessibility encouraged AST among children. After adjusting for all demographic and socioeconomic attributes, among parental perception variables, only perceived presence of maintained sidewalks and presence of shade-casting street trees around the home were associated with AST in the model.

## 4. Discussion

In this study, we statistically explored both objective and perceived home-neighborhood factors on whether or not children choose AST (i.e., walked to/from school) among a cohort of 749 children aged 12–14 years. Novel street connectivity measures from space syntax were used to measure street network design in the home-environments (i.e., 800 m buffers). Children’s and parents’ perceptions of walking barriers, assessed from a cross-sectional sample and obtained from questionnaires, were also investigated. By analyzing the objectively measured built-environment features from space syntax and simultaneously combining this with attitudinal factors, we have provided important new insights into their effect on children’s AST.

### 4.1. Exploratory Findings

We found that a majority of children (76%) walked to/from school. This rate is in sharp contrast to the findings of North American [95] or Australian studies [96]. For example, Su et al. [20] reported that only 20% of students walked to school from a sample of 4338 students in 10 communities within the Los Angeles metropolitan area, while this rate was 34% for children walking to school in Victoria, Australia [97]. The relatively higher share of walking for the school trips in our study is consistent with a report on İstanbul, which showed that 71% of the trips were conducted on foot for the school journey [98]. This is mainly due to the fact that the vast majority of Turkish children attend schools closest to their homes. According to the rules of Ministry of National Education, children are enrolled in the elementary schools closest to their home-addresses [99]. However, there are always exceptions, for example, parents are able to enroll their children in any school they want by either registering their children’s residence in the district where the school is located or by registering the address where they work [100].

Consistent with past research, our findings also showed that distance was a commonly reported barrier to active travel for both parents and children [101,102]. When actual network distances between participants’ homes and schools were measured separately for children and parents who considered perceived distance being a barrier for walking, it was found that the distance perceived by children as “too far” was almost 30% higher than that considered by parents. Encouraging the use of mixed modes (i.e., public transport) or park-and-stride as alternatives to private car travel for the school journey, especially among children residing beyond walking distance to their school, might help integrate active transport as a part of the school journey and hence may contribute to higher levels of physical activity among children [103]. Safety concerns from parents stemmed mostly from issues related to violence and harassment (28.5%), and to a lesser degree, traffic concerns (17.3%). We also found that parental safety concerns were associated with lack of children density within the neighborhood and has been reported elsewhere. Timperio et al. [104] found that children living in neighborhoods with many other children were more likely to actively commute to school since they may have more opportunities to walk to school in the company of other children. This suggests a need to create pedestrian-friendly streets and neighborhoods to increase the potential for socialization for children, which may in return elevate positive parental perceptions regarding safety. The notion has been posited by Moore [105]. He suggested that urban street spaces are necessary for children because unlike recreation areas, they provide easily accessible areas that are interesting and allow for play and socialization. Similarly, initiatives such as Play Streets, which provide opportunities for children all ages to play outdoors, close their homes [106], can be implemented to help ease parental concerns, stimulating an active environment.

### 4.2. Regression Outputs

The results from our nominal logistic regression model results showed that objectively measured home-neighborhood factors obtained from space syntax were more strongly correlated with AST than perceived factors. Notably, after adjusting for demographic and socioeconomic characteristics, increased neighborhood street connectivity, measured by metric reach, was significantly related to increased AST. This is in line with earlier findings, where a positive association between street connectivity and walking to school was discovered [107,108,109]. More importantly, the novel street connectivity measures used in this study showed that apart from street density, directional accessibility (i.e., more direct routes), as measured by directional reach, was also associated with increased AST. Our findings suggest that increasing street network density and reducing direction changes within a 10-minute walking range of homes can encourage AST. Thus, retrofitting neighborhood design to promote connectivity has strong potential to facilitate walking to and from school, and would inherently have positive health benefits for children by way of promoting their AST.

Our empirical findings also showed, albeit modestly, that parental attitudes concerning the built-environment also contribute to children’s AST behavior. In detail, we found that children were more likely to walk to/from school when their parents thought their neighborhood sidewalks were maintained and shade-casting street-trees were present. This finding is supportive of the findings of previous studies, which identified parental perception of sidewalk as the strongest predictor of independent mobility among children [110] and demonstrated that the presence of good sidewalks in the neighborhood was associated with more physical activity in children [111]. However, our finding also suggests that the mere presence of continuous sidewalks or street-trees is not sufficient to encourage AST; sidewalks should be regularly maintained, and shade-casting trees should be planted along the streets to provide a sense of enclosure, which has shown to increase walking mode shares [112]. Hence, our overall finding argues for sidewalk improvements which correspond with many of the principles of “complete streets” [113]. This has health implications for children too. For example, Lovasi et al. [114] observed that street trees were associated with a lower prevalence of early childhood asthma.

Unlike the findings from many past studies indicating an inverse association between parents’ socioeconomic status and walking to school [35,115], our model results showed that children of parents with more education and who owned one car (i.e., high SES) tended to walk to/from school. Although this finding is somewhat counterintuitive, it finds support from Panter et al. [116] and Timperio et al., [104], who also reported children living in more deprived areas were less likely to walk to school. It is unclear why this pattern has emerged, but it is possible that low-income families are displaced to more deprived locations with reduced walkability (i.e., poorly connected street network with inadequate pedestrian-oriented infrastructure) and may be utilizing mass-transit as their primary mode to school. Accordingly, parental concerns of neighborhood safety might be higher in this group due to lack of pedestrian-friendly built-environment conditions, leading to preventing their children from walking to/from school. Thus, improving areas where walkability is low may be required to promote AST.

### 4.3. Policy and Design Implications

Findings presented in this study provide important evidence for planners and policy-makers to effectively design neighborhoods aimed to encourage AST among children. The use of this mode appears to be important for children’s school travel in İstanbul. Our results point to the selected space syntax measures of street design as vital correlates to children’s AST at the trip-origin. We believe that a walkable environment which provides enhanced accessibility is a necessary condition for promoting AST among children. Further, our findings also suggest that minimizing the walking distance to school by designing a connected street network with relatively direct connections between origin-destination pairs is critical. Increased AST is expected to have both individual (reduced risks of childhood obesity) and environmental (promotion of sustainable cities) benefits. A complementary approach to support AST may be improvement in the walking infrastructure. As an example, maintaining sidewalks and street trees so as to provide ‘enclosure’. These interventions may decrease parental concerns when deciding if their child should use AST. Our findings may also help to inform policy relating to school siting. Changing school-siting policies to locate schools in neighborhoods where distances are walkable and street network layouts are designed to create a dense, well-connected, and walkable environment near the home may encourage AST mode choices. Our findings also highlighted that parental concerns of street-level environmental conditions are of importance. To address parental safety concerns on crime and traffic, implementing “School Streets”—in which the streets surrounding the school are restricted to traffic during school drop-off and pick-up times to prioritize walking and cycling—can be listed as a policy strategy aimed to promote AST [117]. This intervention, which has been in use for several years, especially in European Countries, and has had a renewed focus recently with COVID-19, supports AST by emphasizing a healthier lifestyle and reducing road danger [118].

### 4.4. Study Limitations and Strengths

Limitations include cross-sectional data, which makes it impossible to infer causal relationships. A logical avenue for a future study is to conduct a similar survey among this group for longitudinal analysis. An additional phenomenon we did not account for is residential self-selection. Parents supportive of their children walking might choose to live in more walkable neighborhoods. Hence, future research should control for these issues by implementing a case-control approach. Second, our case study sample is representative of dense urban areas, where distances to school are relatively shorter and the environment is more walkable as compared to their rural counterparts. Thus, further research that includes both urban and rural locations is needed to test the generalizability of our results. Third, streetscape qualities (e.g., presence of traffic lights and crossings) around homes and walkability of the school neighborhoods are also important elements that may explain commuting behavior, but which were not included in the analysis. The school-neighborhood built environment was not studied as the parental survey only asked about the perceptions of parents regarding their home-neighborhood. The inclusion of school-neighborhood conditions along with home-neighborhood attributes into the analysis can capture the full journey to school since the former can also influence AST [94]. Fourth, other types of land-use measures (e.g., land-use mix, number of openings onto the street) may also be included to measure the associations of land-use diversity on AST. Further research can include these variables, which may lead to stronger associations with environmental attributes. Moreover, spatial autocorrelation of AST near the home or school was not accounted for, so the model may be mis-specified; however, it likely did not affect the directionality of the reported coefficients. Finally, we did not ask the children their reasons for walking to/from school. Future work can investigate built environment facilitators, as well as barriers, in influencing the decision to AST, which would lead to a more comprehensive understanding of factors at play.

A key strength of this study is that it integrated novel street connectivity measures from space syntax. Introduction of these measures into AST literature is important because these refined measures can affect the interface between urban design and planning by informing design decisions about alternative street alignments, fronting, and orienting developments or alternative ways of introducing interventions on selected streets (such as calming measures). This study has also contributed to the literature due to its focus on Turkey, which has received minimal attention in the past: most of the past research has focused on North America, Canada, and Australia. Although the study area was confined to the Anatolian part of İstanbul, participants were drawn from 93 diverse neighborhoods that varied substantially in education and street network connectivity. Additionally, a nationally representative study found that 72% of children typically walked to school [119]. This is similar to the rate of active commuting identified in the present sample, suggesting it may be representative of the Turkish population.

## 5. Conclusions

Promoting modal shifts to more active modes of school travel (e.g., walking) among children has strong potential for decreasing obesity rates and facilitating sustainable urban environments. To encourage AST among children, it is essential to identify consistent correlates of children’s commuting behavior. This study addressed the evidence gap by examining objective built-environment features from space syntax and the perception of home-environment attributes by parents. Moreover, the results of this study demonstrated new and important factors contributing to children’s AST.

The potential role of built-environment interventions around the home (within 10 minutes of walking distance) has been further supported by the findings of this study. Particularly, the novel urban design metrics from space syntax—metric and directional reach—demonstrated that changes in the spatial structures to increase connectivity may encourage AST for children within a feasible walking distance between home and school. The findings also showed that socioeconomic status matters: children from higher-income families (i.e., higher car ownership) were more likely to walk to/from school. Hence, households with lower socioeconomics, who may have been displaced to more deprived locations with reduced walkability (i.e., low street connectivity and inadequate pedestrian-oriented infrastructure) emerged as a promising target group for intervention to promote AST. For example, street networks within such urban areas should be densified (e.g., by creating footways or alleyways within neighborhoods that lack them) and walking infrastructure (e.g., well-maintained sidewalks) should be improved. These interventions can help create more walkable environments, which will promote real and perceived safety, and thus increase AST among children.

Children’s travel mode decisions are governed by a variety of complex factors, including objective conditions and parental perceptions of the built-environment. Therefore, a combination of both objective and subjective environmental factors should continue to be examined in concert to direct planning and policy interventions which encourage AST mode choices. In addition to facilitating “walking to school”, local urban conditions that are conducive to this mode will provide for a safe, sustainable, and healthy environment.

## Figures and Tables

**Table 1:** Description of variables used in the study.

Variable	Description
School travel mode	
Walk to and from school (both ways)	1 = Yes 0 = No
Walk to or from school (either way)	1 = Yes 0 = No
Demographic characteristics	
Children’s gender	1 = Male 2 = Female
School grade of the children	6th, 7th, 8th grades
Socio-economic characteristics	
Paternal educational status	High (university degree) vs. low
Maternal educational status	High (university degree) vs. low
Number of private cars owned by the household	1 = 0, 2 = 1, 3 = more than 1
Parental perceived home-neighborhood	5-point Likert scale, strongly disagree to strongly agree
Objective home-neighborhood (800 m buffer)	
Residential use	Continuous variable in (m^2^)
Retail use	Continuous variable in (m^2^)
Recreational use	Continuous variable in (m^2^)
Metric reach (800 m)	Continuous variable in (m)
2-Directional reach (10°)	Continuous variable in (m)
Factors discouraging walking to school	
Reasons for not actively commuting (students)	Multiple choice and open ended
Reasons for not allowing child to AST (parents)	Multiple choice and open ended

**Table 2:** Composition of study participants (*n* = 1802) by district and school; number of children per school, average age and number of participating children per gender group and walk mode shares per school.

District	School	Gender	# of Children	Mean Age	Walk Mode Share (% Walking Both Ways)	Walk Mode Share (% Walking Either Way)
Üsküdar (inner city, *n* = 364)	S01	M	50	13.1	44.8	59.4
		F	47	13.1		
	S02	M	43	13.2	67.0	72.5
		F	44	13.1		
	S03	M	41	13.0	62.5	80.7
		F	47	13.2		
	S04	M	46	13.1	69.7	85.4
		F	46	13.1		
Kadıköy (inner city, *n* = 495)	S01	M	32	12.9	76.3	89.5
		F	44	13.3		
	S02	M	42	13.5	63.6	75.3
		F	35	13.4		
	S03	M	46	13.2	48.4	52.7
		F	44	13.0		
	S04	M	39	13.3	34.6	39.7
		F	39	13.2		
	S05	M	52	14.0	60.0	61.9
		F	54	14.0		
	S06	M	36	13.1	64.7	73.5
		F	32	13.1		
Kartal (outer city, *n* = 307)	S01	M	44	13.1	69.8	78.1
		F	52	13.1		
	S02	M	57	13.2	61.2	72.4
		F	41	13.2		
	S03	M	60	13.1	83.2	92.0
		F	53	13.1		
Ümraniye (outer city, *n* = 301)	S01	M	60	14.2	76.6	89.7
		F	47	14.0		
	S02	M	45	13.1	81.1	90.6
		F	61	13.1		
	S03	M	47	14.0	79.5	97.7
		F	41	14.1		
Ataşehir (peripheral suburb, *n* = 335)	S01	M	40	13.1	77.1	83.1
		F	43	13.0		
	S02	M	51	13.2	78.1	87.5
		F	46	13.1		
	S03	M	41	14.1	37.2	44.2
		F	45	14.0		
	S04	M	35	13.9	71.4	88.6
		F	34	14.0		

**Table 3:** Commuting mode shares among the sample.

Mode of Commuting	Commuting to School		Commuting from School	
	*n*	%	*n*	%
car	266	14.8	135	7.5
school shuttle	262	14.4	261	14.5
public transport	50	2.8	69	3.8
cycle	0	0	0	0
walk	1224	68	1337	74.2
Total	1802	100	1802	100

**Table 4:** Attributes associated with children’s walk mode choice (either way), for children living within 1600 m of their schools. *n* = 749.

Attributes	Description	Estimate	SE	Wald X^2^	*p*-Value
CHILD					
gender	male	0.06	0.11	0.31	0.58
	female	–	–	–	–
HOUSEHOLD					
parental education *	yes	0.24	0.12	3.95	0.05
	no	–	–	–	–
**number of cars (1–0)**	categorical	0.67	0.25	14.35	0.01
number of cars (≥2–1)	categorical	0.70	0.38	14.35	0.06
LAND-USE					
**residential use (m^2^)**	continuous	−0.00	0.00	20.17	0.00
**retail use (m^2^)**	continuous	−0.00	0.00	15.34	0.00
recreational use (m^2^)	continuous	0.00	0.00	0.01	0.94
STREET DESIGN					
**2-Directional reach (10°)**	continuous	0.23	0.10	4.79	0.03
**Metric reach (800 m)**	continuous	0.38	0.08	20.32	0.00
STREETSCAPE					
presence of continuous sidewalks around the home	agree	−0.11	0.17	0.39	0.53
	other	–	–	–	–
**presence of maintained sidewalks around the home**	agree	0.24	0.12	4.12	0.04
	other	–	–	–	–
presence of trees around the home	agree	−0.00	0.16	0.00	0.96
	other	–	–	–	–
**presence of shade-casting trees along the roads**	agree	0.32	0.16	4.21	0.04
	other	–	–	–	–
walking is easy around the home	agree	−0.05	0.12	0.14	0.71
	other	–	–	–	–
STREET NETWORK LAYOUT					
presence of mainly short intersections (≤100 m) around the home	agree	−0.18	0.12	2.12	0.15
	other	–	–	–	–
presence of many alternative streets to travel between origins and destinations around the home	agree	−0.11	0.15	0.51	0.47
	other	–	–	–	–

* both parents having a college degree or above. Note: Statistically significant attributes (*p* < 0.05) are in bold. District ID was used as a fixed effect.

## Data Availability

The data presented in this study are available on request from the corresponding author. The data are not publicly available due to confidentiality.
